# Lafora Disease: A Ubiquitination-Related Pathology

**DOI:** 10.3390/cells7080087

**Published:** 2018-07-26

**Authors:** Maria Adelaida García-Gimeno, Erwin Knecht, Pascual Sanz

**Affiliations:** 1 Department of Biotechnology, Polytechnic University of Valencia, 46022 Valencia, Spain; agarcia@ibv.csic.es; 2 Centro de Investigación Príncipe Felipe, 46012 Valencia, Spain; eknecht@cipf.es; 3 Centro de Investigación Biomédica en Red de Enfermedades Raras, (CIBERER), 46010 Valencia, Spain; 4 Instituto de Biomedicina de Valencia, Consejo Superior de Investigaciones, Científicas, 46010 Valencia, Spain

**Keywords:** Lafora disease, ubiquitination, malin, E3-ligase, proteostasis, glycogen accumulation

## Abstract

Lafora disease (LD, OMIM254780) is a rare and fatal form of progressive myoclonus epilepsy (PME). Among PMEs, LD is unique because of the rapid neurological deterioration of the patients and the appearance in brain and peripheral tissues of insoluble glycogen-like (polyglucosan) inclusions, named Lafora bodies (LBs). LD is caused by mutations in the *EPM2A* gene, encoding the dual phosphatase laforin, or the *EPM2B* gene, encoding the E3-ubiquitin ligase malin. Laforin and malin form a functional complex that is involved in the regulation of glycogen synthesis. Thus, in the absence of a functional complex glycogen accumulates in LBs. In addition, it has been suggested that the laforin-malin complex participates in alternative physiological pathways, such as intracellular protein degradation, oxidative stress, and the endoplasmic reticulum unfolded protein response. In this work we review the possible cellular functions of laforin and malin with a special focus on their role in the ubiquitination of specific substrates. We also discuss here the pathological consequences of defects in laforin or malin functions, as well as the therapeutic strategies that are being explored for LD.

## 1. Introduction

Lafora disease (LD, OMIM254780, ORPHA501) is a rare and fatal form of progressive myoclonus epilepsy (PME). The term PME comprises a group of neurological disorders characterized by the presence of focal and generalized seizures, myoclonus and progressive neurodegeneration, accompanied with cerebellar signs and dementia [1,2,3]. Among PMEs, LD is unique because of the rapid neurological deterioration of patients and the appearance of insoluble glycogen-like (polyglucosan) inclusions, named Lafora bodies (LBs), in the brain and peripheral tissues [4,5]. LD was first described in 1911 by the Spanish neurologist Gonzalo Rodríguez Lafora [4], who reported the presence in post mortem preparations of intense dark inclusions that he called “amyloid bodies”. In early adolescence, LD manifests with generalized tonic-clonic seizures, myoclonus, absences, drop attacks, and visual hallucinations. Additional initial symptoms comprise confusion, depression, and intellectual decline. As the disease progresses, seizures become worse and a rapid dementia with apraxia (inability to perform purposive actions), aphasia (impairment in the production and comprehension of speech), and visual loss ensues. Gradually, LD patients enter in a vegetative state with continuous myoclonus, requiring tube feeding and artificial respiration. Eventually they die, usually within the first decade from onset of the first symptoms, from status epilepticus or aspiration pneumonia [6,7]. Apart from the clinical presentation, LD is diagnosed by the presence of LBs in axillar samples, although due to high rate of false negatives, at present the confirmation of the diagnosis comes from genome sequencing of the genes related to the disease (see below). LD occurs worldwide, but is relatively more frequent in Mediterranean countries (e.g., Spain, France, Italy), Northern Africa, the Middle East, and the Southern regions of India [7].

In this work, we review the possible cellular functions of laforin and malin, the two main proteins defective in LD, with a special focus, since malin is an E3-ubiquitin ligase, on their role in the ubiquitination of specific substrates. We also discuss here the pathological consequences of defects in laforin or malin functions, as well as the therapeutic strategies that are being explored for LD.

## 2. Genetic Basis of the Disease

LD is a very rare disease with a prevalence of less than 4 patients per 1,000,000 individuals [7]. It is an autosomal recessive disorder caused, almost always, by mutations in two genes: *EPM2A*, encoding laforin, a glucan phosphatase, and *EPM2B/NHLRC1*, encoding malin, an E3-ubiquitin ligase [8,9,10]. According to the recent literature, around 50–60% of LD patients carry mutations in the *EPM2A* gene, whereas 40–50% carry mutations in the *EPM2B* gene [6,7]. In both cases, all possible types of genetic modifications, e.g., deletions, insertions, frameshifts, and nonsense and missense mutations, occur in patients’ genomes. Patients can be homozygous for a particular genetic modification or compound heterozygous carrying a different modification in each allele of the same gene. There is a database that recollects all modifications described so far: http://projects.tcag.ca/lafora. Until June 2018, this database identified 105 modifications in the *EPM2A* gene (3 polymorphisms, 32 deletions/insertions/frameshifts, 13 nonsense, and 57 missense mutations) and 85 modifications in the *EPM2B* gene (5 polymorphisms, 26 deletions/insertions/frameshifts, 8 nonsense, and 46 missense mutations). In both genes, modifications are scattered throughout the complete sequence.

The geographic distribution of the mutations varies depending on the country. While in some countries, like Spain, *EPM2A* mutations are more prevalent, in others, like Italy, mutations in the *EPM2B* gene are more common [11]. In the case of *EPM2A*, one of the most frequent mutations is R241X, whereas in the case of *EPM2B*, P69A is the most frequent mutation described in the literature [7,12].

Patients with mutations in laforin or malin are neurologically and histologically indistinguishable [12], suggesting that these two proteins operate through common physiological pathways. LD is considered by many authors as a clinically homogeneous disorder [7]. However, in some cases, there is a late-onset or a slow progression of the disease. In these cases, it is assumed either that the mutation present in *EPM2A* or in *EPM2B* only partially affects the function of the corresponding protein, or that the presence of genetic or environmental modifiers influences the regular progression of the disease [13,14,15,16].

## 3. Laforin

**Figure 1:**
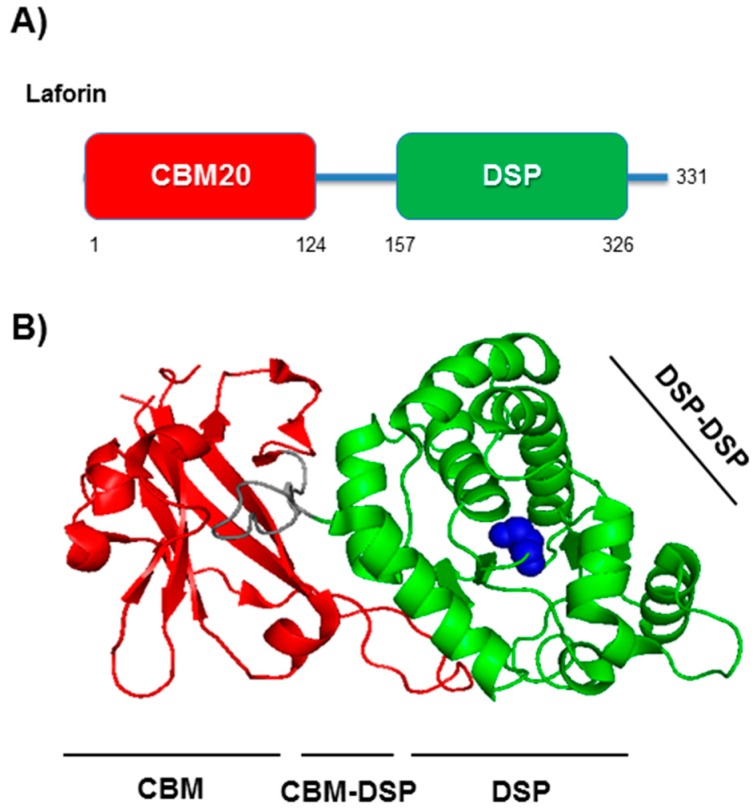
Structural view of laforin. (**A**) Diagram of the different structural domains in laforin; CBM20, carbohydrate binding module type 20; DSP, dual specificity phosphatase domain. Numbers indicate the location of these domains in the laforin protein sequence. (**B**) Crystal structure of a laforin monomer according to Ref. [30]. CBM domain is coloured in red and DSP domain in green. The different regions where the pathogenic mutations cluster are indicated (CBM, CBM-DSP, DSP and DSP-DSP dimerization region). The catalytic Cys residue is coloured in blue.

Laforin is encoded by the *EPM2A* gene, containing four exons and located on the chromosome 6q24 of the human genome, which produces a major mRNA with an ORF of 993 nts, according to the ENSEMBL database. It is expressed in all tissues, but particularly in brain, skeletal muscle, heart, and liver [17]. Laforin is a protein of 37 kDa (331 amino acids) with a carbohydrate binding module (CBM) at the N-terminus (residues 1–124) and a dual specificity phosphatase domain (DSP) at the C-terminus (residues 157–326) (Figure 1A). According to the Carbohydrate-Active enZYmes (CAZY) database [18], the CBM domain of laforin belongs to one of the best characterized CBM families, CBM20 [19], being this domain also present in glucosyltransferases and glucotransferases from bacteria to plants [20,21,22]. In the case of laforin, its CBM domain allows laforin to bind glycogen and complex carbohydrates (e.g., amylopectin) [19,23,24].

Laforin contains also a C-terminal DSP (Figure 1A). This domain is present in the large protein tyrosine phosphatase (PTP) family of cysteine-dependent phosphatases, which utilizes a conserved CX5R motif to hydrolyze phosphoester bonds [25,26]. In the case of laforin, the catalytic residue is Cys266. The DSP family includes phosphatases that may dephosphorylate non-proteinaceous substrates [26,27]. Regarding laforin, its DSP domain affords it to dephosphorylate complex phosphorylated carbohydrates such as glycogen and amylopectin [28,29]. Laforin is the only human phosphatase with a CBM domain present in the same polypeptide chain as the catalytic phosphatase domain [26]. It is assumed that the ability to bind carbohydrates, mediated by the CBM domain, allows laforin to bind, using its DSP domain, to the substrate that has to be dephosphorylated. For this reason, mutations in the CBM domain impair the phosphatase activity of laforin with similar strength than mutations in the DSP domain [17].

The laforin crystal structure was recently described [30] (Figure 1B). It consists of two laforin molecules in the asymmetric unit, each containing a CBM and a DSP domain. Both laforin molecules form an antiparallel dimer, being connected by the DSP domain of each monomer. In addition, the CBM domain is in close connection with the DSP domain, forming an integrated tertiary structure, which was crucial to understanding the possible function of laforin in the dephosphorylation of glycogen. Moreover, it provided a blueprint to localize the different pathogenic mutations, which were found to be clustered in the CBM domain, the CBM-DSP interface, the DSP domain and the DSP-DSP dimer interface [30] (Figure 1B).

## 4. Malin

**Figure 2:**
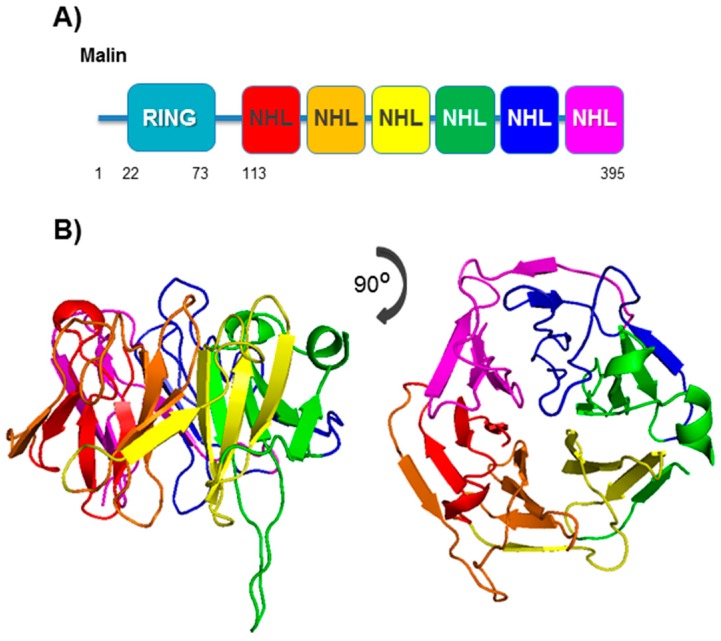
Structural view of malin. (**A**) Diagram of the different structural domains in malin; the position of the RING (Really Interesting New Gene) and NHL (NCL1, HT2A and LIN-41 containing) domains is indicated. (**B**) In silico modeling of NHL domains. The protein sequence of malin containing the NHL domains was submitted to the ESypred3D server and modeled using the structure of *M. tuberculosis* PknD (PDB:1rwl) as template. Each NHL domain is stained with a different color and side and top views are on the left and right panels, respectively.

Malin is encoded by the single exon *EPM2B* gene, located on chromosome 6q22.3 of the human genome, which produces a major mRNA with an ORF of 1185 nts, according to the ENSEMBL database. It is expressed in brain, heart, skeletal muscle, liver, and pancreas [10]. Malin, a protein of 42 kDa (395 amino acids), is an E3-ubiquitin ligase with a RING finger domain of the C3HC4 type at the N-terminus (residues 22–73) and six NHL domains (also present in NCL1, HT2A and LIN-41 proteins) at the C-terminus (residues 113–395) (Figure 2A). The NHL domains are predicted to fold into a β-propeller structure, which mediates protein–protein interactions [31] (Figure 2B). Since malin encodes an E3 enzyme, LD can be considered as a form of pathology associated, at least in part, with the ubiquitin system. However, even when several models have been proposed, the precise molecular mechanisms whereby the malin defect results in LD are still unknown.

**Figure 3:**
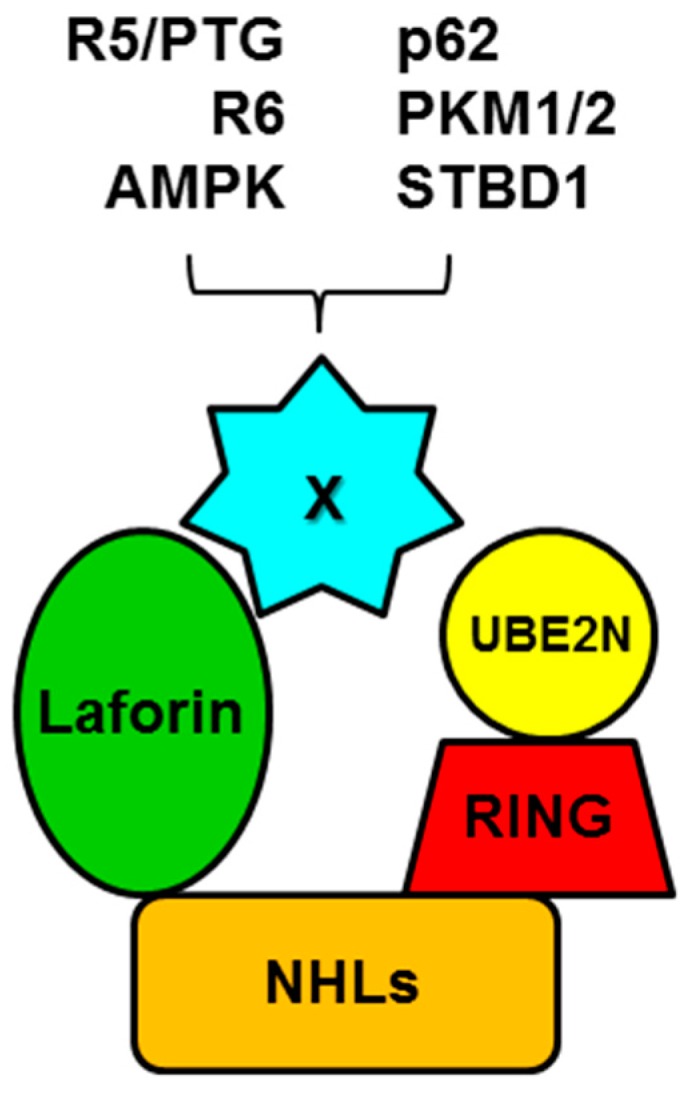
Schematic view of the laforin-malin complex and some of its interactors. Malin is depicted with its two domains, RING and NHLs, and interaction with one of its specific E2-conjugating enzymes, UBE2N, is also shown. X refers to putative substrates (see text for details).

As an E3-ubiquitin ligase, malin is able to ubiquitinate specific substrates. To perform this function, malin interacts with specific E2-conjugating enzymes. Forty-one different E2-conjugating enzymes have been described thus far in the human genome (https://www.genenames.org/cgi-bin/genefamilies/set/102) and in vitro assays have demonstrated that malin is able to interact with UBE2D1, UBE2D2, UBE2D3, and UBE2E1, but not with UBE2C and UBE2L3 [32]. Recently, our group reported that malin interacts physically and functionally with UBE2N but not with UBE2D2 [33] (Figure 3). We also described that malin incorporates both monoubiquitin and polyubiquitin moieties to the corresponding substrates. As ubiquitin itself has seven inner Lys residues (K6, K11, K27, K29, K33, K48, and K63), polyubiquitin chains may be formed by the attachment of a new ubiquitin moiety to one of those Lys residues present in the former ubiquitin. In the case of malin substrates, polyubiquitination is obtained mainly by K63-linked ubiquitins [34], which makes sense with the fact that the E2 UBE2N promotes K63-linked polyubiquitination. The topology of the polyubiquitin chains makes a big difference in the final cellular fate of the protein. For example, K11-linked and K48-linked chains are the main signals to target substrates for proteasomal degradation, while K63-linked chains have principally other functions different from proteasomal degradation, including the degradation of the polyubiquitinated proteins by autophagy and other non-degradative functions [35]. Various proteins have been reported to be substrates of malin, as follows (in brackets the defined topology of the polyubiquitin chains; see also Table 1 and Figure 3): (i) laforin (K63-Ub) [34]; (ii) the glycogenic subunits of PP1 phosphatase R5/PTG (K63-Ub) [34] and R6 (mono-Ub and K63-Ub) [36]; (iii) AMP-activated protein kinase (AMPK), subunits α and β (K63-Ub) [37]; (iv) the autophagy receptor p62 (K63-Ub) [33]; (v) pyruvate kinase isozymes M1 and M2 (PKM1/2) (K63-Ub) [38]; (vi) starch-binding domain containing protein 1 (STBD1) (K63-Ub) (unpublished results); and (vii) the component of the Wnt signaling pathway dishevelled2 (K48- and K63-Ub) [39].

A phylogenetic study suggested that malin gene is exclusively found in vertebrate genomes and in the cephalochordate *Branchiostoma floridae* [43]. This distribution does not correlate with that of laforin, which is conserved in all vertebrates, in a reduced group of protozoans and in two invertebrate genomes, but which is absent in most protozoans and in invertebrate genomes such as yeast, fly, and worm [43]. These results suggest that, at least in those organisms where malin is absent, laforin may have malin-independent functions [43].

The same report indicates that malin shares an evolutionary relationship with TRIM32, an E3-ubiquitin ligase that belongs to the tripartite motif-containing (TRIM) protein family [43]. Both malin and TRIM32 exhibit a similar modular structure, containing a functional RING domain at the N-terminus and six NHL domains at the C-terminus. Although malin lacks the B-box and coiled-coil domains present in TRIM32 [43], both proteins show some functional redundancies. Thus, when overexpressed in cell culture systems, TRIM32 is capable of ubiquitinating some malin substrates (e.g., R5/PTG, AMPK subunits), but with a different chain topology. This redundancy is not reciprocal since specific TRIM32 substrates (e.g., PIASy or dysbindin) are not ubiquitinated by malin. Cumulatively, the phylogenetic studies suggest that malin initially evolved from an ancestral TRIM gene and developed a particular E3-ubiquitin ligase activity [43].

When overexpressed, malin is prone to aggregate. The co-chaperone CHIP (C-terminus of Hsc70 Interacting Protein) and the chaperone Hsp70 stabilize malin [44]. In fact, laforin, malin, and CHIP form a ternary complex, which is involved in the HSF1-mediated response to heat shock [45].

## 5. Laforin and Malin form a Functional Complex

Patients with mutations in laforin or malin are neurologically and histologically indistinguishable [12]. Therefore, how can defects in two proteins with so different enzymatic activities lead to similar pathological phenotypes? The answer to this question came when our group and others found that laforin and malin interact physically and form a functional complex [32,34,46]. Therefore, pathology could not only arise from mutations that affect laforin and malin activity, but also from mutations that simply impair the interaction of both proteins to form the complex. 

In this complex laforin could, for example, recognize and recruit putative substrates to be ubiquitinated by malin for degradation (Table 1 and Figure 3). In fact, most malin substrates directly interact with laforin but not with malin. Laforin does not require its glucan phosphatase activity for this recruiting activity, since a laforin C266S mutant, which lacks phosphatase activity, interacts with malin and its substrates even better than laforin WT [34]. We have also described that phosphorylation of the laforin S25 residue by AMPK improves the interaction of laforin with malin [47]. This observation connects the energy status of the cell with the activity of the laforin-malin complex, which would be maximal under energy deprivation conditions that activate AMPK. Finally, degradation of laforin is promoted by malin ubiquitination [32], thus providing an additional regulation of the malin-dependent ubiquitination of other substrates. In this regard, it is worth saying that that laforin protein levels decrease if cells are grown in the absence of glucose [48], probably as a consequence of a more active laforin-malin complex.

## 6. Subcellular Localization of Laforin and Malin

Overexpression studies with tagged version of laforin suggested that laforin has a cytoplasmic distribution, mostly associated with endoplasmic reticulum markers [49,50]. However, under stress, such as nutrient starvation and heat shock, laforin translocates to the nucleus, returning again to the cytoplasm when the cells recover from these conditions [42,45]. Attempts to define the subcellular localization of endogenous malin have been unfortunately hampered by the lack of a sensitive anti-malin antibody. Overexpression studies using tagged-malin forms have suggested that malin has a nuclear localization, which does not change under stress conditions [45]. Therefore, the nuclear localization of malin and, under certain conditions, of laforin suggests a regulatory function of both proteins in the cell nucleus. One of these possible nuclear functions is the regulation of HSF1 (Heat Shock Factor 1), a transcription factor involved in the expression of genes related to the heat shock response [45].

## 7. Pathophysiological Consequences of a Dysfunctional Laforin-Malin Complex

### 7.1. Altered Regulation of Glycogen Synthesis

**Figure 4:**
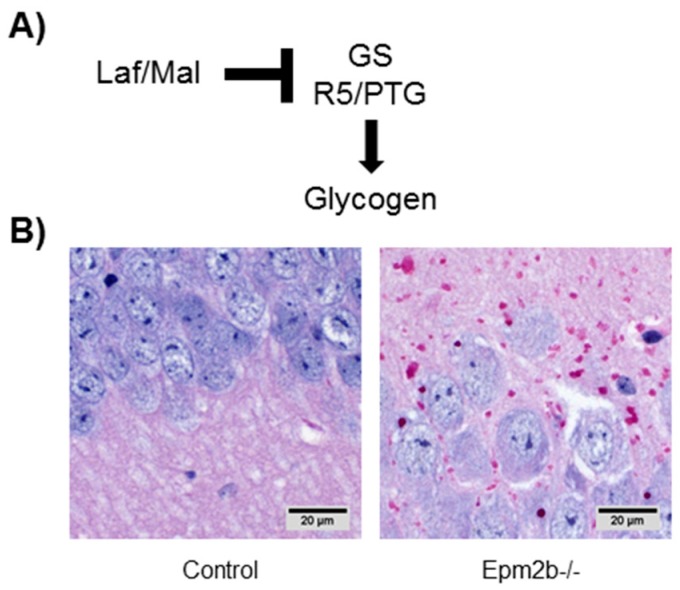
The hallmark of LD is the accumulation of LBs. (**A**) Diagram indicating the possible role of the laforin-malin complex regulating glycogen synthesis; GS: glycogen synthase. (**B**) Samples from the hippocampus of 12 months old control and Epm2b-/- mice (lacking malin) were stained with periodic acid Schiff reagent (PAS staining), which colors polysaccharide granules in red.

As mentioned above, the hallmark of LD is the accumulation in brain and peripheral tissues of poorly-branched glycogen-like inclusions named LBs. These structures also contain higher levels of phosphomonoesters on the glucose residues [51,52]. Both properties (less branching and higher phosphate content of glycogen) make LBs largely insoluble. One of the first identified substrates of the laforin-malin complex was a glycogenic regulatory subunit of protein phosphatase 1 named R5/PTG (Protein Targeting to Glycogen) [34,40,41]. The function of R5/PTG (PPP1R3C) is to recognize phosphorylated substrates involved in glycogen homeostasis, such as glycogen synthase and glycogen phosphorylase, to recruit them to the PP1 phosphatase, and trigger their dephosphorylation. This will cause the activation of glycogen synthase (since it is inactive in its phosphorylated form) and the inactivation of glycogen phosphorylase (active in its phosphorylated form). Thus, the net effect of the activity of R5/PTG-PP1 holoenzyme is an increase in glycogen synthesis [34,40,41]. On the other hand, the laforin-malin complex recognizes R5/PTG and targets it for inactivation, most probably by proteolysis, and in this way, it could participate in the negative regulation of glycogen synthesis (Table 2). In the presence of a dysfunctional laforin-malin complex, R5/PTG would be more active, leading to an enhanced glycogen synthesis, which is compatible with the accumulation of LBs (Figure 4). In addition to R5/PTG, other PP1 glycogenic targeting subunits such as GL (PPP1R3B) and R6 (PPP1R3D) have been described as additional substrates of the laforin-malin complex ([36,41]). Moreover, other glycogen-related proteins, such as glycogen synthase itself and the glycogen debranching enzyme (AGL), have been also described as substrates of the laforin-malin complex [40,42]. In this case, the presence of a dysfunctional laforin-malin complex would lead to higher levels of glycogen synthase and AGL, which would result in the formation of higher levels of poorly branched glycogen species (Figure 4) (Table 2). In all these cases, the laforin-malin complex mainly promotes a K63-linked polyubiquitination (see above, Section 4), which would cause the degradation of the affected proteins (Table 1) mainly by autophagy, but also by proteasomes when this modification is combined with some other type of ubiquitin linkages.

Although the results described above have been confirmed by different labs, they are largely based on results obtained under conditions of overexpression of laforin and malin in cell cultures using recombinant proteins. In contrast, the levels of some glycogen related proteins, such as glycogen synthase or R5/PTG, do not increase in animals of 3–6 months of age, as it would be expected by the lack of a functional laforin-malin complex [51,52,67]. However, non-degradative functions also exist for a K63-linked polyubiquitination and we hope to reconcile these contradictory observations in the future.

**Figure 5:**
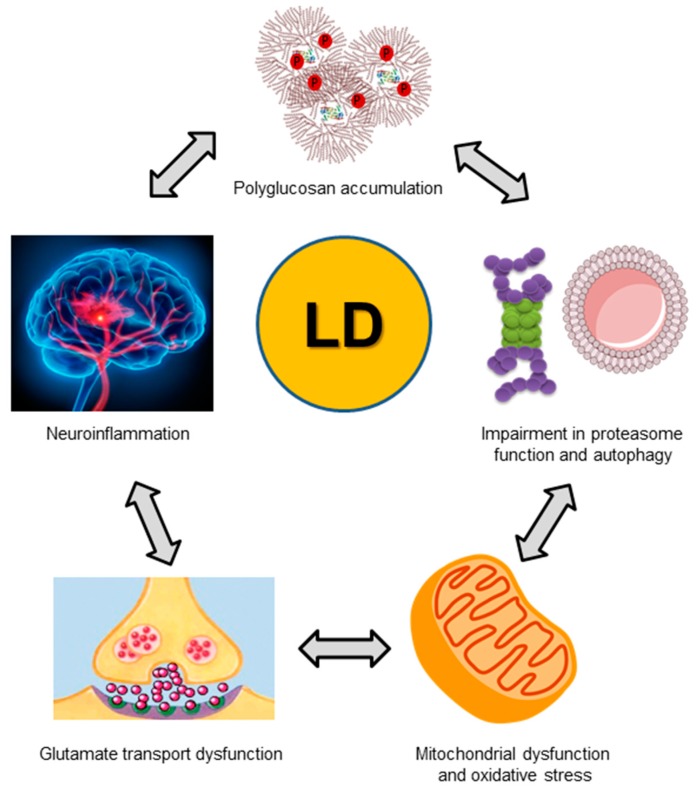
Some of the identified alterations in LD. As indicated in the text, LD is characterized by the accumulation of poorly-branched hyperphosphorylated glycogen-like inclusions (polyglucosans, top). In addition, several defects in cellular proteostasis have been described: impairment in proteasome and autophagy activities (middle right), mitochondrial dysfunction (bottom right), etc. Glutamate neurotransmission is also affected in LD (bottom left panel), and clear signs of brain inflammation (top left panel) have been described in mouse models of the disease.

In any case, the animal models of LD clearly show an accumulation of insoluble glycogen-like structures (polyglucosans) in different tissues (Figure 5). These LBs are resistant to the action of α-amylase, indicating that they cannot participate in providing energetic substrates under energy demand. This is particularly important in the brain, where glycogen breakdown (glycogenolysis) has been described as a key process to obtain the energy required to maintain critical processes for neuronal activity, such as K^+^ and glutamate uptake [68,69,70]. If glycogenolysis is prevented, then the cells will become more hyperexcitable, lowering the threshold for the appearance of seizures, which are quite relevant in the pathophysiology of LD [71].

### 7.2. Altered Homeostasis of Glucose Transporters

Glucose enters the cell through specific glucose transporters. In the case of brain, the main glucose transporters are GLUT1 and GLUT3, expressed predominantly in astrocytes and neurons, respectively. The amount of these transporters at the plasma membrane, which is regulated by cycles of endocytosis and exocytosis, determines the efficiency of glucose uptake. It has been described [48] that overexpression of laforin and malin induces the endocytosis of these transporters, whereas in the absence of these proteins the glucose transporters are constitutively located at the plasma membrane (Table 2). These authors further suggested that an increase in glucose uptake in LD would raise the levels of glucose-6P (an allosteric activator of glycogen synthase), thus resulting in increased glycogen synthesis. However, the mechanism of action of the laforin-malin complex on the glucose transporters is still poorly understood. It could act either directly on them or indirectly on some related proteins, such as the kinase SGK1 that regulates their homeostasis [54] (Table 2).

### 7.3. Altered Regulation of Proteostasis

In addition to carbohydrate moieties, LBs contain up to 6% of proteinaceous material, including glycogen synthase, laforin, chaperones, autophagic components, proteasome subunits, advanced glycation end products, etc. [58,62,72]. Most of these products are polyubiquitinated, suggesting an attempt of the cells to eliminate LBs by the usual protein clearance mechanisms, namely proteasomes and autophagy. An activation of the unfolded protein response pathway, which eventually would lead to increased endoplasmic reticulum stress, has been also reported in LD [58,59], probably as a consequence of the impairment in protein degradation. In this regard, it has also been described that laforin interacts physically with the chaperone Hsp70 and helps to decrease the toxicity of the unfolded proteins [73]. Therefore, all these results suggest a possible role of the laforin-malin complex in regulating cellular proteostasis (Table 2). In fact, it has been suggested that in the absence of laforin or malin, there is a decrease in the activity of proteasomes [59,61] and an impairment in autophagy, which likely occurs at the initial step of autophagosome formation [62,63,74]. However, the precise molecular mechanisms of action of laforin and malin in these processes are still unknown.

Moreover, due to the autophagy impairment, processes that require a functional autophagy machinery are also affected in cellular and animal models of the disease. In this sense, we have recently described a defect in mitophagy, most likely due again to defects in autophagosome formation [65] (Table 2). Autophagy impairment could affect negatively glycogen degradation, as one of the glycogenolytic pathways involves lysosomes, but we have recently observed in astrocytes from mouse models of LD that the main glycogen degradation involves cytosolic glycogen phosphorylase and not lysosomal α-glucosidase [75].

Finally, we have also reported that oxidative stress is affected in the absence of a functional laforin-malin complex [60], since we found, in cellular and animal models of LD, higher levels of reactive oxygen species (ROS) and of oxidative stress products. This defect is probably due to an altered mitochondrial function and also to dysregulated expression of antioxidant enzymes and decreased activity of enzymes involved in the detoxification of ROS [60] (Table 2).

### 7.4. Altered Homeostasis of the Astrocytic Glutamate Transporter

We and others have described that in animal models of LD there is a transformation of astrocytes into GFAP+ reactive astrocytes [55,56]. In addition, there is also an increase in reactive IBA1+ microglia [55,56] (Table 2). These changes indicate a general reprograming in the expression of different proteins to allow the affected cells to cope with the stress conditions. One of the processes that is affected due to the reactivity of the astrocytes is the function of the glutamate transporters [76]. Glutamate is the main excitatory neurotransmitter in the CNS. It is released by the presynaptic neurons upon arrival of an action potential and binds to specific receptors at the post-synaptic neuron in order to elicit an excitatory signal. As this signaling event must be transient, glutamate has to be removed from the synaptic cleft to prevent an overstimulation of the post-synaptic neuron. This re-uptake is carried out by the astrocytic terminals that surround synapsis [77,78,79,80]. Astrocytes, but not neurons, express glutamate transporters that take up glutamate in these terminals. The main transporter carrying out this function is the Excitatory Amino Acid Transporter 2 (EAAT2; GLT-1 in mice), a trimeric protein that is located at the plasma membrane in the astrocyte terminals. This transporter is subjected to rounds of endocytosis and exocytosis at the plasma membrane regulated by environmental conditions [81,82,83,84]. We have recently described that the homeostasis of the glutamate transporter EAAT2 is affected in cellular models lacking laforin or malin [53] (Figure 5). Although the total levels of the protein were similar to those found in healthy cells, the fraction of the protein present at the plasma membrane was reduced in LD cellular models. We investigated the possible cause of this alteration and found that the pattern of ubiquitination of the EAAT2 transporter changed in the absence of laforin or malin. These changes in ubiquitination probably account for the altered distribution of the EAAT2 transporter in LD cellular models (Table 2). This is an important point, since dysfunction in glutamate uptake would increase the levels of glutamate in the synaptic cleft, leading to excitotoxicity, a condition that is related with the appearance of seizures [85].

### 7.5. Enhanced Inflammatory Reaction

The presence of reactive astrocytes and microglia is associated with pro-inflammatory conditions. In agreement with this, we have described that in the brain of animal models of LD there is an increase in the levels of pro-inflammatory mediators such as TNFα, IL-6 and IL-1β, chemokines, cytokines, and other inflammatory markers [57]. The establishment of these inflammatory conditions may alter the viability of neurons and could lead to neurodegeneration (Table 2 and Figure 5).

What triggers the inflammatory process? The existence of an interrelationship between epilepsy and inflammation has been widely documented [86,87,88]. One possibility is that an initial seizure event could trigger the inflammatory response in astrocytes and microglia, and this process is amplified along the age of the animals. Another possibility is that the accumulation of LBs is the initial event that triggers inflammation. In line with this latter possibility, we have recently described that astrocytes are the main brain cellular type that accumulates polyglucosan inclusions in animal models of LD [75]. Perhaps the presence of these inclusions inside the astrocyte triggers a stress reaction in the form of a release of pro-inflammatory mediators, chemokines and cytokines, which would initiate the inflammatory reaction. Microglia may also contribute in the same way, as we also observed microglial cells loaded with polyglucosan inclusions [75].

### 7.6. Altered Wnt Signaling Pathway

It has been reported that malin interacts with dishevelled2, a key component of the Wnt signalling pathway. Dishevelled2 is a cytosolic phosphoprotein whose activation results in the inactivation of the degradation pathway of β-catenin, resulting in the accumulation of this protein and in its translocation to the nucleus, where it activates the transcription of a specific set of genes. Overexpression of malin enhances degradation of dishevelled2 and inhibits Wnt signalling through the downregulation of β-catenin protein levels and, thus, of the expression of β-catenin target genes. Since malin enhances both K48- and K63-linked ubiquitination of dishevelled2, its degradation could occur through both proteasomes and autophagy [39] (Table 2). As Wnt signalling is involved in synaptic differentiation, synaptic plasticity, and neurogenesis, the increased levels of dishevelled2 due to a loss of function of malin may alter these processes via an increased Wnt signalling.

### 7.7. Altered Function of Processing Bodies

Processing bodies (PBs) are cytoplasmic structures that house the RNA decay machinery (mRNA-decapping enzyme 1A Dcp1a, activators of decapping and the major 5′-3′ exoribonuclease XRN1). It has been reported that malin, when overexpressed, is recruited to the PBs, where it interacts with Dcp1a and promotes its degradation via the ubiquitin proteasome system (Table 2). Since depletion of malin results in increased levels of Dcp1a, it was suggested that the dysregulation in mRNA metabolism may underline some of the symptoms in LD [66].

## 8. Treatment Strategies

There is currently no proven therapy for LD. The initial symptoms of seizures and epilepsy are treated with regular anti-epileptic drugs (AEDs; e.g., valproate), but patients soon become resistant to them [7]. Based on the characteristics of the pathophysiology of LD, several approaches are currently being studied, as follows.

### 8.1. Treatments Based on Decreasing Glycogen Synthesis

If the hallmark of the disease is the accumulation of insoluble poorly branched glycogen-like structures (LBs), it seems logical to think that any action aimed to decrease glycogen synthesis could be beneficial. This assumption was elegantly demonstrated by different authors, which created mice models combining the absence of laforin or malin and of R5/PTG or glycogen synthase. Obviously, these mice synthesized no or only small amounts of glycogen, because of the lack of key regulators of glycogen synthesis, but in addition and more interestingly, these double knock-out mice showed no symptoms of LD [55,89,90,91]. These results suggest that strategies aimed to decrease glycogen synthesis are beneficial for treating LD. In this sense, at present, different treatments are being tested, including the administration of anti-sense oligonucleotides to decrease the expression of glycogen synthase or of chemical compounds that inhibit the enzyme. These strategies are still in their infancy and time is needed to assess if any of them become suitable as an effective therapy for LD.

### 8.2. Treatments Based in Recovering Proteostasis and Decreasing Inflammation

Since proteostasis is affected in LD, other groups have analyzed the possibility of using different drugs to improve proteostasis. In this sense, since autophagy is impaired in LD, we and others checked whether inducers of autophagy, such as inhibitors of the autophagy regulator mTORC1, could be beneficial for treating this disease. However, the use of rapamycin, everolimus or temsirolimus had no effect ([92] and unpublished results). In contrast, we recently described some beneficial effects after administration of animal models of LD with either 4-phenylbutyrate (a molecular chaperone) or metformin (an indirect activator of AMPK) [56]. LD mice treated with either of these compounds presented a lower number of LBs in different areas of the brain, reduced appearance of reactive astrocytes and microglia, and better performance in functional behavioral tests. Since the effects of metformin were the most marked of both, we obtained an orphan drug designation of it for the treatment of LD, approved both by the European Medicines Agency (EMA) and by the U.S. Food and Drug Administration (FDA). Metformin has a complex mechanism of action. Firstly, it indirectly activates AMPK, since it inhibits mitochondrial complex I, leading to reduced production of ATP, increased AMP/ATP ratio and, thus, activation of AMPK, which is a stimulator of autophagy and has recognized anti-inflammatory and neuroprotective properties [93,94,95]. Secondly, different AMPK-independent mechanisms have been described for metformin [96,97]. Perhaps a combination of all these mechanisms could account for the beneficial effects of metformin in LD. In any case, these findings open the way for the search of more potent AMPK activators that could be used in LD.

Finally, additional compounds for the treatment of LD have been tested by other authors. One example is provided by sodium selenate [98], a compound with antioxidant and anti-inflammatory properties which protects from neurodegeneration and from the appearance of reactive gliosis.

### 8.3. Treatments Based in Decreasing Excitatory Neurotransmission

It has been recently described that the use of perampanel, an antagonist of post-synaptic AMPA receptors, has beneficial effects for LD in terms of decreasing the frequency of seizures and improving neurocognitive processes [99,100]. These results suggest that therapeutic strategies aimed to decrease excitatory neurotransmission could be beneficial in LD.

## 9. Perspectives

LD is a devastating pathology that leads to the death of the patients after around ten years from the beginning of the first symptoms. In recent years, many data have accumulated trying to understand the molecular basis of the pathophysiology of LD. Although several relevant discoveries have been made, the primary defect that triggers LD is still controversial. For example, we still do not understand why LD cells accumulate more glycogen than normal cells and why this polysaccharide is poorly branched and hyperphosphorylated. We also do not know how proteostasis is affected, how neuroinflammation is initiated, or how epilepsy is triggered. We hope that in the near future, we could provide answers to these yet unsolved questions. In the meantime, different strategies are being explored to obtain a treatment that slows down the progression of the disease and diminishes its symptoms.

## Figures and Tables

**Table 1:** Substrates of the E3-ubiquitin ligase malin.

Protein	Function	Topology of Ubiquitin Labeling	References
R5/PTG (PPP1R3C)	Glycogen metabolism	K63-Ub	[34,40,41]
R6 (PPP1R3D)	Glycogen metabolism	Mono-Ub and K63-Ub	[36]
GL (PPP1R3B)	Glycogen metabolism	Unknown	[41]
Glycogen synthase	Glycogen metabolism	Unknown	[40]
Debranching enzyme AGL	Glycogen metabolism	Unknown	[42]
Starch-binding domain containing protein 1	Glycogen metabolism	K63-Ub	Unpublished results
Pyruvate kinase, isozymes M1 and M2	Glycolysis	K63-Ub	[38]
AMPK, subunits α and β	Energy sensor	K63-Ub	[37]
Laforin	Unknown, ancillary protein of malin?	K63-Ub	[32]
p62	Autophagy receptor	K63-Ub	[33]
Dishevelled2	Wnt-β-catenin signaling	K48- and K63-Ub	[39]

**Table 2:** Affected cellular components and processes in Lafora disease.

Cellular Component/Process	General Defect in Lafora Disease	References
Glycogen synthesis	Accumulation of poorly branched glycogen-like inclusions (Lafora bodies)	[34,36,40,41,42]
Glutamate transport	Impaired astrocytic glutamate uptake	[53]
Glucose transporters	Increased glucose uptake due to altered homeostasis of GLUT1 and GLUT3	[48,54]
Inflammation	Increased reactive astrocytes and microglia. Increased expression of pro-inflammatory markers, cytokines and chemokines	[55,56,57]
Wnt signaling	Increased Wnt signaling	[39]
ER-stress	Increased sensitivity to unfolded protein response	[58,59]
Oxidative stress	Increased oxidative stress	[60]
Proteasomes	Inhibition of proteasome activity	[59,61]
Macroautophagy	Impairment in the initial steps of macroautophagy	[62,63,64]
Mitochondria	Dysfunctional mitochondria	[60,65]
Processing bodies	Dysregulation of RNA metabolism	[66]
